# Leaf status and environmental signals jointly regulate proline metabolism in winter oilseed rape

**DOI:** 10.1093/jxb/erz538

**Published:** 2019-12-06

**Authors:** Younes Dellero, Vanessa Clouet, Nathalie Marnet, Anthoni Pellizzaro, Sylvain Dechaumet, Marie-Françoise Niogret, Alain Bouchereau

**Affiliations:** 1 INRA, UMR 1349 Institut de Génétique, Environnement et Protection des Plantes, INRA, Agrocampus Ouest, Université de Rennes 1, Rennes, France; 2 Plateau de Profilage Métabolique et Métabolique (P2M2), INRA-IGEPP and INRA-BIA, Le Rheu, France; 3 University of Exeter, UK

**Keywords:** *Brassica napus*, fluxes, osmotic stress, *Δ*^*1*^*-PYRROLINE-5-CARBOXYLATE SYNTHASE* (*P5CS*), *PROLINE DEHYDROGENASE* (*ProDH*), proline, regulation, senescence

## Abstract

Proline metabolism is an essential component of plant adaptation to multiple environmental stress conditions that is also known to participate in specific developmental phases, particularly in reproductive organs. Recent evidence suggested a possible role for proline catabolism in *Brassica napus* for nitrogen remobilization processes from source leaves at the vegetative stage. Here, we investigate transcript levels of *Δ*^*1*^*-PYRROLINE-5-CARBOXYLATE SYNTHASE* (*P5CS*) and *PROLINE DEHYDROGENASE* (*ProDH*) genes at the vegetative stage with respect to net proline biosynthesis and degradation fluxes in leaves having a different sink/source balance. We showed that the underexpression of three *P5CS1* genes in source leaves was accompanied by a reduced commitment of *de novo* assimilated ^15^N towards proline biosynthesis and an overall depletion of free proline content. We found that the expression of *ProDH* genes was strongly induced by carbon starvation conditions (dark-induced senescence) compared with early senescing leaves. Our results suggested a role for proline catabolism in *B. napus,* but acting only at a late stage of senescence. In addition, we also identified some *P5CS* and *ProDH* genes that were differentially expressed during multiple processes (leaf status, dark to light transition, and stress response).

## Teaser

Illumination conditions and acquisition of source status by winter oilseed rape leaves jointly impact on proline levels, expression levels of proline metabolism-related genes, and associated metabolic fluxes.

## Introduction

Winter oilseed rape (*Brassica napus* L.) is a major oleaginous crop ranked the third most essential source of plant oil in the world. Considering the agroecological transition and climate change contexts, there is a need to maintain crop seed yield and quality under fluctuating environments and to reduce chemical inputs. However, oilseed rape is highly demanding for mineral nitrogen (N) inputs (150–250 kg N ha^–1^) and characterized by a poor N use efficiency, with only 50% of the N absorbed by plants being present in seeds (Rossato *et al.*, 2001; Rathke *et al.*, 2005). Therefore, optimization of N management by oilseed rape under reduced N input conditions and/or during abiotic stress is a promising target for future crop improvement (Masclaux-Daubresse *et al.*, 2010; Bouchet *et al.*, 2016). In plants, N is taken up first from the soil by roots through nitrate and ammonia transporters. Nitrate is preferentially transported in the xylem to be assimilated in source leaves and then further reallocated to sink tissues in the form of amino acids (Tegeder and Masclaux-Daubresse, 2018). Although oilseed rape is characterized by a strong N uptake efficiency at the vegetative stage, it has a low N use efficiency, mainly due to a low N remobilization efficiency (Malagoli *et al.*, 2005). At the vegetative stage, N remobilization in oilseed rape occurs essentially between leaves according to their specific sink/source balance, through regulated sequential senescence processes, and involves a complex proteolytic degradation system (Girondé *et al*., 2015). Indeed, a complete metabolic reprogramming is triggered to help protein degradation and successive steps of proteinogenic transamination of amino acids into glutamine for N recycling and reallocation (Chrobok *et al.*, 2016). The specific overexpression of glutamine synthetase in midveins during natural leaf senescence and under N limitation conditions suggests that glutamine is a major vector of N for N remobilization through the phloem in oilseed rape (Orsel *et al.*, 2014). However, others amino acids may also contribute to N remobilization, depending on the physiological leaf status and environmental agroclimatic conditions. In *B. napus*, previous studies pointed out the importance of proline metabolism for stress response under N and water depletion, as well as a potential role in N remobilization from source tissues at the vegetative stage (Albert *et al.*, 2012; Faës *et al*., 2015; Zhang and Becker, 2015; Clément *et al*., 2018).

Proline is considered as a compatible osmolyte that accumulates widely in the plant kingdom during varying abiotic stress conditions (i.e. drought, high salinity, high light, heavy metals, and phosphate starvation, and in response to biotic challenges) (Trotel *et al.*, 1996; Slama *et al.*, 2015; Aleksza *et al.*, 2017). It can act as an osmoprotective and osmoregulating compound by chaperoning macromolecules under low water environment and readjusting the internal cellular osmotic potential (Verslues and Sharma, 2010). In plants, proline biosynthesis from glutamate takes place in the cytosol and the chloroplasts, and is initiated first by the NADPH-dependent delta-1-pyrroline-5-carboxylate synthase (P5CS) (Szabados and Savoure, 2010). This enzyme produces a glutamate semialdehyde that is spontaneously converted to pyrroline-5-carboxylate (P5C). Then P5C is further reduced to proline by the NADPH-dependent P5C reductase (P5CR). Proline biosynthesis is directly connected to the NADPH/NADP^+^ balance and is often seen as a sink for reducing power under stress-disturbed redox status, thereby lowering reactive oxygen species (ROS) production (Ben Rejeb *et al.*, 2014). In Arabidopsis, two *P5CS* genes were identified with specific functions: *P5CS1* is induced by drought and osmotic stress while *P5CS2* is required during seed maturation and expressed in response to biotic stress (Savouré *et al.*, 1995; Yoshiba *et al.*, 1995; Fabro *et al.*, 2004; Székely *et al*., 2008). In *B. napus*, a total of six *P5CS1* genes have been identified (Wang *et al.*, 2014). Previous analysis revealed that a *P5CS1* (*BnaA05g05760D)* and a *P5CS2* (*BnaA09g35230D*) were involved in proline production during water shortage, salt stress, or polyethyleneglycol (PEG) stress (Xue *et al.*, 2009; Albert *et al.*, 2012). Proline catabolism occurs in mitochondria via the sequential action of proline dehydrogenase (ProDH) and P5C dehydrogenase, which convert proline successively to P5C then to glutamate (Schertl *et al.*, 2014). Two *ProDH* genes exist in Arabidopsis, with ProDH1 playing an essential role for proline-dependent oxidation/respiration in Arabidopsis mitochondria (Cabassa-Hourton *et al.*, 2016). Recently, the response of the single mutants *prodh1* and *prodh2* and the double mutant *prodh1prodh2* to a prolonged darkness treatment was analysed. The results showed that *ProDH1* and *ProDH2* were both involved in the proline-dependent mitochondrial respiration during dark-induced senescence (Launay *et al.*, 2019). In *B. napus*, six *ProDH1* and two *ProDH2* genes were identified by phylogenetic analysis with parental ancestors (*B. rapa* and *B. oleracea*) (Faës *et al*., 2015). *BnaA&C.ProDH1a* and *BnaA&C.ProDH1b* genes were very highly expressed in leaves and roots, whereas *BnaA&C.ProDH1c* genes were almost exclusively expressed in reproductive organs. *BnaProDH2* genes were mainly expressed in flowers, roots, and senescent leaves. Analysis of promoter–β-glucuronidase (GUS) lines showed a strong expression of *BnaProDH2* genes in vascular tissues, proposing a role for proline degradation, which would provide energy for phloem transport (Faës *et al*., 2015). Proline biosynthesis can also derive from ornithine through the action of ornithine δ- or α-aminotransferase, but these alternative pathways were shown to be irrelevant for proline biosynthesis during stress conditions (Funck *et al.*, 2008).

Regulation of proline metabolism under optimal or stress conditions is well documented and usually involves the modulation of the *P5CS*/*ProDH* balance at the transcriptional level, through either an abscisic acid (ABA)-dependent or an ABA-independent pathway (Zarattini and Forlani, 2017). Under osmotic/salt/drought/and multiple other abiotic stress conditions, induction of *P5CS* gene transcription allows plants to acclimate to the stress by accumulating proline and scavenging reducing power (Yoshiba *et al.*, 1995; Signorelli and Monza, 2017). During the post-stress metabolic recovery phase, induction of *ProDH* gene transcription plays a key role in proline recycling, as it is a readily available source of N and reducing power for mitochondrial metabolism (Sharma and Verslues, 2010; Dobrá *et al*., 2011). Additionally, light and dark conditions can affect proline metabolism in Arabidopsis. *P5CS* gene transcription is promoted during the light period and repressed during dark periods, whereas *ProDH* transcript levels show the opposite behaviour (Hayashi *et al.*, 2000; Abrahám *et al*., 2003). Prolonged exposure to darkness (dark-induced senescence) was also reported to cause an activation of *ProDH* transcription and a depletion of proline content, mediated by activationof sucrose-dependent *bZIP* factors (Dietrich *et al.*, 2011; Launay *et al.*, 2019). At the reproductive stage, decreasing water potential during flower senescence in *Rosa hybrida* L. can trigger the accumulation of proline through increasing *P5CS* transcription (Kumar *et al.*, 2008; Zhang and Becker, 2015).

Considering the role of *ProDH* genes during dark-induced senescence in Arabidopsis (Launay *et al.*, 2019), and the specific expression of *ProDH2* genes in the midveins of source tissues in *B. napus* (Faës *et al*., 2015), proline catabolism might contribute to phloem-dependent N remobilization processes in source leaves of *B. napus*. Such a role would demonstrate that proline metabolism is a novel target for the improvement of N remobilization efficiency in crops. In this work, we addressed the question of the role of proline metabolism in the source leaves at the vegetative stage in the economically important plant crop *B. napus*. We systematically analysed the expression of the *P5CS* and *ProDH* genes related to the biosynthesis and degradation of proline, and measured the associated net proline fluxes (synthesis versus degradation) using ^15^N labelling experiments. Considering that the allopolyploid nature of oilseed rape can confer multiple possibilities of sub-/neofunctionnalization of proline metabolism-associated gene copies, we also analysed their expressions during stress conditions (osmotic stress and dark-induced senescence), in order to delimit the regulatory genes of interest. The analysis of the *BnaP5CS/BnaProDH* transcript balance with respect to the modifications of net proline metabolic fluxes revealed that proline catabolism was weakly activated according to the sink/source balance of the leaves. The depletion of proline content in source leaves was associated with a decrease in the net proline biosynthesis flux and was correlated with decreased expression of five *BnaP5CS1* genes. In addition, we identified some *BnaP5CS* and *BnaProDH* genes that were differentially expressed during multiple processes (leaf status, dark to light transition, and stress response).

## Materials and methods

### Plant material and growth conditions

Seeds of oilseed rape *B. napus* genotype Aviso were obtained from our ‘Bracysol’ biological resource centre. After a 3 d germination step on soaked blotting paper, seedling were transferred in 4 litre pots filled with a non-fertilized commercial substrate (Falienor, reference 922016F3). Plants were grown in a 6 m^3^ growth chamber under a 14 h light/10 h dark cycle (22 °C/16 °C) in ambient air (65% and 80% humidity) with a photosynthetically active radiation of 100 µmol photons m^–2^ s^–1^ at the top of the canopy. A commercial fertilized solution (Liquoplant Bleu, 2.5% N, 5% P, 2.5% K) was applied twice a week on plants. All experiments were performed on plants grown for 60 days after sowing (60 DAS), possessing 14 leaf ranks, annotated from the bottom to the top (L3 to L16). The two oldest leaves (L1 and L2) had already fallen off, confirming that the remobilization processes between leaves holding a different sink/source balance were operating.

### Chlorophyll and soluble protein contents

Relative chlorophyll quantification in SPAD units was achieved using a non-destructive chlorophyll SPAD-502 meter (Minolta). For protein extraction, samples were freeze-dried for 72 h then ground to a fine powder. Soluble proteins were extracted in a buffer containing 20 mM citrate, 160 mM Na_2_HPO_4_ (pH 6.8), and a pinch of polyvinylpyrrolidone by a 15 min incubation step at 1500 rpm. After a 30 min centrifugation step at 4 °C, proteins from the supernatant were quantified using the Bradford reagent with BSA as the standard.

### Nitrogen and carbon percentage

N% and C% were determined on 1 mg of lyophilized plant powder and the Dumas combustion method with an NA1500CN Fisons instrument (Thermoquest, Runcorn, Cheshire, UK) analyser.

### Leaf discs assays

Leaf discs (0.8 cm^2^) were randomly punched with a cork-borer in both laminas of the leaves. For ^15^N labelling experiments, leaf discs were floated for 4 h in a 6-well microplate filled with a buffer containing 10 mM MES-KOH (pH 6.5), 10 mM ^15^NH_4_Cl (99%), or [^15^N]l-proline (98%). From each leaf, two time points were sampled (0 and 4 h) in order to calculate ^15^N incorporation into amino acids before and after the experiment. For hyperosmotic experiments, leaf discs were floated on a buffer containing 5 mM HEPES (pH 6), 1.5 mM CaCl_2_, and 10 mM KCl supplemented or not with 400 g of PEG 6000 per kg of H_2_O. This PEG concentration was calculated accordingly to Michel and Kaufman’s equation to provide a –1.88 MPa osmotic shock to leaf discs (Michel and Kaufmann, 1973). After 18 h of stress, leaf discs were transferred to a hypo-osmotic medium (the same medium without PEG) for 6 h to allow tissue rehydration. All incubations were performed under continuous light in an orbital shaker at 70 rpm. For each harvested time point, leaf discs were rinsed three times with a neutral buffer (without PEG, ^15^NH_4_Cl, or [^15^N]l-proline) and dried for 30 s on a clean tissue before being frozen in liquid nitrogen and stored at –80 °C. For dark-induced senescence experiments, leaf discs were floated on water and the control experiment was done by incubating the microplate under a classical light/dark cycle (14 h/10 h). Complete dark conditions were mimicked by covering the microplate with a thick black plastic bag.

### Amino acid content and ^15^N labelling

Prior to metabolite extraction, samples were freeze-dried for 72 h, allowing measurement of the dry weight. Metabolites were extracted with a mixture of MTBE/MeOH/H_2_O, except that dl-3-aminobutyric acid (111 µM final concentration in a 0.9 ml MeOH/H_2_O fraction) was used as internal standard for amino acid quantification (Salem *et al.*, 2016). A 200 µl aliquot of the remaining MeOH/H_2_O fraction was evaporated using a SpeedVac concentrator at 35 °C for 2 h. Amino acids were resuspended in 50 µl of ultrapure water, and 5 µl were derivatized using an AccQTag derivatization kit (Waters) following the manufacturer’s procedures. Separation and UV detection of amino acids was performed on an Acquity UPLC-DAD (Renault *et al.*, 2010). Amino acids were quantified using external calibration curves for each amino acid and normalized with the internal standard. For ^15^N experiments, isotopologues (M+0, M+1, M+2, M+3, M+4) from AccQTag-derivatized amino acid were analysed by MS by coupling a triple quadrupole spectrometer (Waters) to the analytical system. The electrospray source was settled in positive mode and each isotopologue was tracked with the Selected Ion Recording function. The settings for the mass spectrometer electrospray source were as follow: 4,4 kV capillary voltage, 35 V cone voltage, 150 °C cone temperature, 450 °C desolvatation temperature, and 900 l h^–1^ N flow for desolvatation. Derivatization with AccQTag added ~20% of artificial M+1 enrichment for each amino acid, which was corrected for each sample using the M+1 enrichment observed in ^15^N-free external standards. Considering an amino acid with *n* atoms of N, true ^15^N labelling (%) for each isotopologue *x* was calculated as:

$$\begin{array}{l} {}_{}^{15}N\;\;\;labelling\;\;\;(\;\%\;)=\frac{\;\;\;[M+x]}{\overset{n}{\underset{x=0}{\sum }}\;\;\;[M+x]} \end{array}$$

Net ^15^N labelling for each isotopologue was calculated by subtracting the *M*+*x* labelling (%) from T0 of the experiment (natural abundancy). Net ^15^N incorporation for each amino acid was defined as the absolute quantity of net ^15^N detected in each amino acid from leaf discs after 4 h of ^15^N labelling. The commitment of [^15^N]amino acids in the protein fraction was too low to be detected in our experiments.

### Identification of *P5CS* genes and phylogenetic analysis

*P5CS* genes were identified by blasting *Arabidopsis thaliana P5CS1* (*AT2G39800*) and *P5CS2* (*AT3G55610*) coding sequences retrieved from TAIR database (Berardini *et al.*, 2015), respectively, on the available genomes from ENSEMBL or Phytozome database [*Arabidopsis lyrata* (v.1.0), *B. napus* var *Darmor* (AST_PRJEB5043 v1, assembly 2014), *B. rapa* (Brapa_1.0), *B. oleracea* (BOL), *Oryza sativa* (IRGSP-1.0), *Eutrema salsugineum* (v1.0), and human (GRCh38.p12)]. Sequence alignments and phylogenetic analysis were performed with MEGAX software (Tamura *et al.*, 2013). The evolutionary phylogenetic gene tree was generated with the maximum likelihood method and the nucleotide substitution model of Tamura–Nei, assuming uniform rates among sites (1000 bootstraps).

### Quantitative analysis of transcript levels

Total RNAs were extracted with the NucleoSpin RNA Plus Kit (Macherey Nagel) from frozen or lyophilized tissue ground into a fine powder, following the manufacturer’s instructions. Potential DNA traces were removed with DNase using the RQ1 RNase-free DNase (Promega) according to the manufacturer’s protocol. The absence of DNA contamination in samples was controlled by PCR amplification of the *BnaEF1α* reference gene. RNA integrity was verified by running samples on 1.2% agarose gels. A 1 μg aliquot of total RNA treated with the DNase was reverse-transcribed using oligo(dT)_15_ primer and Goscript reverse transcriptase (Promega) according to the manufacturer’s instructions. For quantuitative PCR (qPCR) experiments, 2 μl of diluted cDNA were mixed with the LightCycler 480 SYBR Green I Master mix (Roche) and primers pairs (see Supplementary Table S2 at *JXB* online). qPCRs were achieved on a Light Cycler LC480 (Roche) under the following conditions: an initial step at 95 °C for 5 min, then 45 cycles of 95 °C for 15 s and 60 °C for 40 s. Two reference genes [*BnaC.UBQ11* (*BnaC04g09150D*) and *BnaC.RibS3* (*BnaC04g13040D*)] were selected for their expression stability in our samples harvested from different conditions (leaf ranks, light/dark conditions, and stress conditions) using the GeNorm method (Vandesompele *et al.*, 2002). Gene expression was normalized using the geometrical mean expression of two reference genes and the efficiency of qPCR primers (Supplementary Table S2). The efficiency of qPCR primers was calculated from a serial dilution of mixed samples from our experiments. Fold change (FC) values were calculated as the ratio of the relative gene expression between two consecutive time points for treated (FC treatment) or control (FC control) leaf discs (T0 and PEG, PEG and PEG+rehydration for osmotic stress; T2d and T4d for dark-induced senescence). Log2 (FC treatment/FC control) corresponded to the log2 value of the ratio (FC treatment/FC control).

### Statistical analysis

The variability of the results is expressed as the mean ±SD of *n* independent biological replicates (*n*=3–6). Means of different leaf ranks were compared together with one-way ANOVA followed by a post-hoc Tukey’s HSD test for multiple pairwise comparison. Means of two treatments (light versus dark; control versus dark-induced senescence; control versus osmotic stress) for each leaf rank were compared using a Student *t*-test (bilateral distribution, equal variance). For statistical analysis of qPCR experiments, relative expression values were log-transformed to obtain normally distributed values. For each test, a *P*-value <0.05 was applied. All statistical analyses were achieved with “R” software.

## Results

### Source status of *B. napus* leaves is associated with a decrease of proline content

**Fig. 1.**
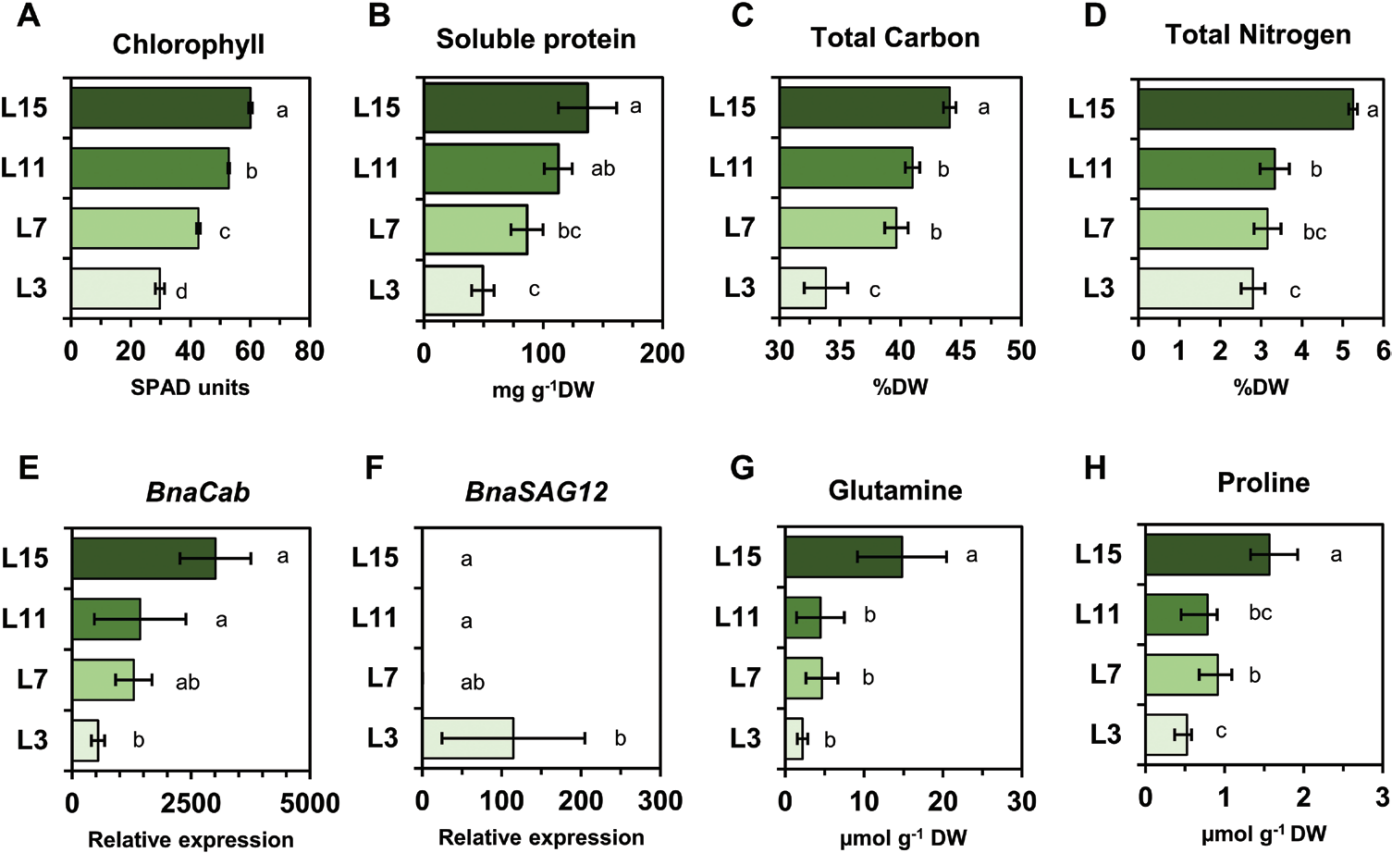
Proline is a potential source of nitrogen remobilized from source leaves at the vegetative stage in *B. napus*. (A) Chlorophyll and (B) soluble protein contents. (C) Total carbon and (D) total nitrogen contents. (E, F) qPCR analysis of *BnaCab* and *BnaSAG12* genes. (G, H) Glutamine and proline contents from four leaf ranks having a different sink/source balance at the vegetative stage (60 DAS). Leaves were harvested 3 h after the beginning of the illumination period. Values are the means ±SD of 3–6 independent biological replicates. Different letters indicate that mean values are significantly different (*P*-value <0.05) between the different leaf ranks. Overall amino acid analysis of the four leaf ranks is listed in Supplementary Table S1. (This figure is available in colour at *JXB* online.)

In order to obtain *B. napus* plants with a gradient of leaf ranks having different sink/source balance, plants were grown for 2 months at the vegetative stage in growth chambers to reach the 16-leaf stage (the two oldest leaves had already fallen off). From this stage, we decided to work on four leaf ranks: a young growing leaf (L15), an immature leaf (L11), a post-mature leaf (L7), and a senescent leaf (L3), essentially functioning as a source tissue (Albert *et al.*, 2012). The status of the four different leaf ranks was checked by quantifying different parameters: chlorophyll and soluble protein contents, total carbon (C) and N contents, the expression of the *Cab/SAG12* reporter genes, and free amino acid contents (Gombert *et al.*, 2006). First, dry weight per leaf area was decreased according to the sink/source balance of the leaves whereas the fresh weight per leaf area was relatively maintained, leading to an increase of the FW/DW ratio between the different leaf ranks (Supplementary Fig. S2). Therefore, all results were expressed on a dry weight basis to compare the different leaf ranks, except for chlorophyll content which was measured per leaf area. Nevertheless, the observed decreased of chlorophyll content per leaf area for the different leaf ranks might still be relevant by normalizing with dry weight (Fig. 1A; Supplementary Fig. S1). Along the leaf nodal ranks, we observed a significant decrease of the content of total soluble proteins and the total C and N contents (Fig. 1B–D). Since amino acids (and therefore proteins) have a high C/N ratio, the decrease of C and N% per dry weight seemed to be attributed to an activation of protein degradation. These results suggest that both C and N remobilization are strongly induced from the L11 leaf. Those observations were also confirmed by the expression levels of *Cab* and *SAG12* genes. *Cab* gene expression was significantly and gradually repressed according to the source status of the different leaf ranks, whereas the *SAG12* gene was significantly expressed in the L3 leaf, mirroring its actual senescent status (Fig. 1E, F). From the overall free amino acid profiles, glutamine content showed a significant decrease, except for L11 and L7 (Fig. 1G; Supplementary Table S1). Such variations of glutamine content between the different leaf ranks is often attributed to the activation of the remobilization processes (proteolytic degradation and amino acids exported as glutamine) (Orsel *et al.*, 2014; Tegeder and Masclaux-Daubresse, 2018). Consequently, the L15 leaf may have a higher ‘sink’ demand compared with its ‘source’ export. Interestingly, although at a lower amplitude, proline content showed statistically significant variations between the different leaf ranks, similarly to glutamine content variations, thereby suggesting a potential role for proline metabolism in nutrient recycling processes in the sources leaves of *B. napus* (Fig. 1H). To summarize, we have identified four leaf ranks with gradually increased levels of source status and gradually decreased levels of proline content from the top to the bottom of the plant axis.

### Decrease of *BnaP5CS1* gene expression in source leaves correlates with a decrease of net proline biosynthesis flux

Next, we decided to analyse the *P5CS* and *ProDH* transcript balance according to the sink/source balance of the leaves and during a dark to light transition. These different conditions may solicit proline metabolism for N reallocation, respiration feeding, and/or redox status adjustment. In order to design gene-specific primers for each copy of *BnaP5CS* (Supplementary Table S2), we performed a genome-wide analysis of *P5CS2* genes in *B. napus* and its progenitors *B. rapa* and *B. oleracea*, using known *P5CS2* coding sequences from the Arabidopsis genome as a query. Our analysis identified four *AtP5CS2* (*Bna.P5CS2*) orthologues, corresponding to two *P5CS2* orthologues in each progenitor genome (*B. rapa* and *B. oleracea*) (Supplementary Fig. S1). We also performed similar work for *P5CS1* in order to localize the genomic region of the six *P5CS1* genes previously identified (Wang *et al.*, 2014). The identified *P5CS1* and *P5CS2* genes from *B. napus*, *B. rapa*, and *B. oleracea* were correspondingly renamed in this study based on the genome to which they belonged or inherited (A for *B. rapa* and C for *B. oleracea*), their chromosome location, and their phylogenetic relationship (Supplementary Fig. S2). Considering *ProDH* genes, a previous work already identified six *BnaProDH1* and two *BnaProDH2* genes and designed specific primers for each gene (Faës *et al*., 2015).

**Fig. 2.**
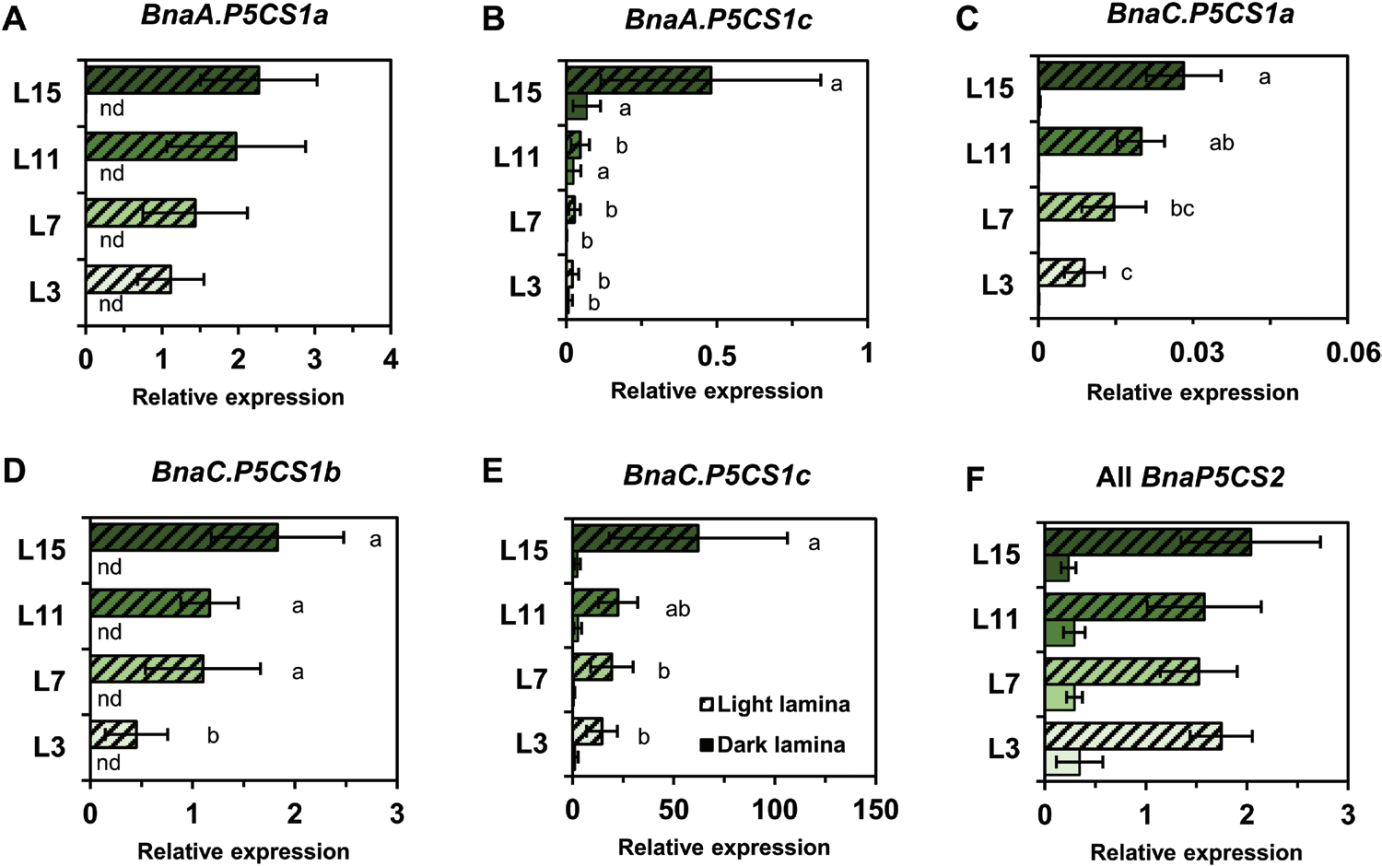
Four *P5CS1* genes are underexpressed in source leaves in *B. napus*. Laminae from four leaf ranks having a different sink/source balance at the vegetative stage (60 DAS) were sampled either 3 h before (dark) or 3 h after (light) the beginning of the illumination period. Expression of each gene copy was normalized relative to two reference genes *UBQ11* and *RibS3*. Relative expression of genes showing no significantly different behaviour during leaf development were pooled together (all *BnaP5CS2* genes). The complete data set for each *BnaP5CS2* gene is available in Supplementary (Fig. S3). Values are the means ±SD of five independent biological replicates. Different letters indicate that mean values are significantly different (*P*-value <0.05) between the different leaf ranks. Comparison of mean values from light and dark conditions were always significantly different for each leaf rank and each gene (*P*-value ≤0.05). (This figure is available in colour at *JXB* online.)

First, *P5CS* transcript levels were quantified relative to the reference genes in the four leaf ranks sampled 3 h before or 3 h after the beginning of the illumination period, in order to evaluate the possibly combined effects of the sink/source balance of the leaves and the natural dark to light transition. Transcript levels for four *BnaP5CS1* genes were significantly decreased in leaves having a very strong source leaf status compared with the L15 leaf, in accordance with the previously observed decrease in proline content (Figs 1F, 2A–E). The expression levels of *BnaA.P5CS1b* are not shown since they were too weak to be repeatedly detected in our samples. *BnaC.P5CS1c* showed the strongest expression levels compared with other *BnaP5CS1* genes, suggesting a preponderant role in controlling endogenous proline levels (Fig. 2B). All *BnaP5CS2* transcript levels were not significantly changed between the different leaf ranks (Fig. 2F; expression levels for each *BnaP5CS2* gene are available in Supplementary Fig. S3). However, all *BnaP5CS* genes (except *BnaA.P5CS1b*) were significantly overexpressed in light conditions compared with dark conditions for all the leaf ranks.

**Fig. 3.**
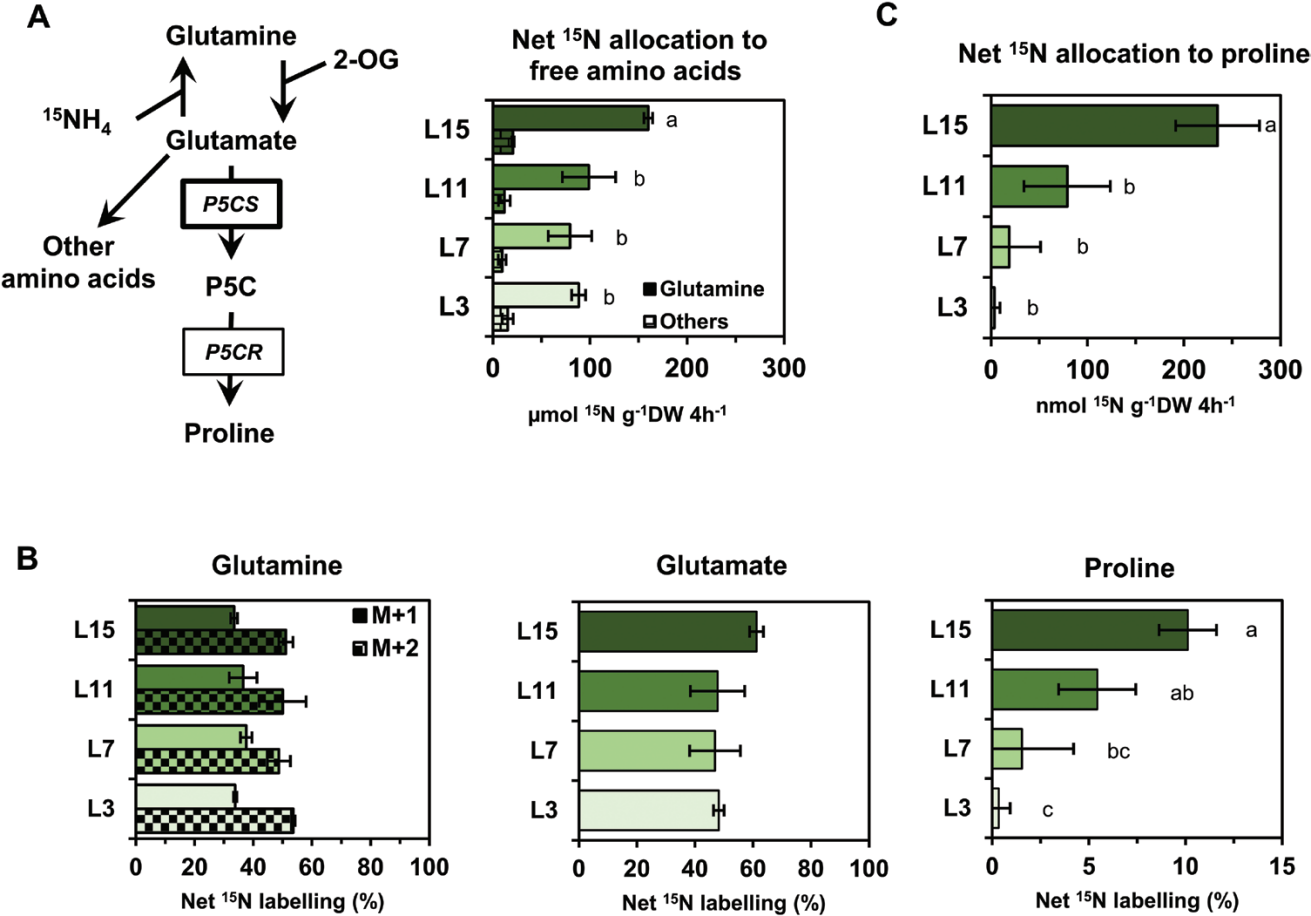
Partitioning of *de novo* assimilated ^15^N towards proline biosynthesis in source leaves correlates with the down-regulation of *P5CS* genes. Leaf discs from four leaves having a different sink/source balance at the vegetative stage (60 DAS) were incubated for 4 h under continuous light with ^15^NH_4_Cl (99%). (A) Metabolic route for ^15^NH_4_ to proline and net ^15^N allocation to free amino acids. (B) Glutamine, glutamate, and proline net ^15^N labelling. (C) Net ^15^N allocation to proline.^15^N labelling detection, and absolute quantification of amino acids were performed by MS and UV detection. Values are the means ±SD of three independent biological replicates. Different letters indicate that mean values are significantly different between the different leaf ranks (*P*-value <0.05). (This figure is available in colour at *JXB* online.)

In order to connect the variations of *BnaP5CS1* transcript levels with the variations of proline content observed in the different leaf ranks, we performed ^15^N labelling experiments on leaf discs isolated from the four leaf ranks. This experiment aimed at comparing *in vivo* the capacity for net proline biosynthesis between the different leaf ranks using the repartition of the ^15^N as a proxy. We took advantage of the leaf capacity to assimilate *de novo* N using ^15^NH_4_ to label glutamate, the major precursor for proline biosynthesis (Fig. 3A). We analysed net ^15^N enrichment and contents of amino acids using an UPLC-TQD system. After 4 h of ^15^N labelling in isolated leaf discs, we found that net ^15^N was in great part allocated to glutamine (Fig. 3A). This allocation was significantly higher in L15 compared with other leaves, showing that L15 has a higher capacity for free ammonia assimilation compared with leaves having a very strong source status. Analysis of ^15^N labelling in glutamine showed similar values for each leaf rank (no statistical differences), with ~30–35% for the M+1 isotopologue and 50–55% for the M+2 isotopologue (Fig. 3B). The ^15^N labelling in the glutamate molecule was also statistically identical between each leaf rank, with values ranging between 48% and 60%. Interestingly, the net ^15^N labelling in the proline molecule was significantly lower between the different leaf ranks (~10% in L15, 7% in L11, and 2–0.5 in L7 and L3). Since the ^15^N labelling in glutamate was similar between the different leaf ranks, our results suggested that the commitment of *de novo* assimilated ^15^N toward proline biosynthesis was lowered in leaves having a very strong source status. To quantitatively evaluate this commitment, we calculated the net ^15^N allocation to proline by assuming that proline commitment toward the protein fraction between the different leaf ranks was negligible (^15^N labelling was undetectable in the protein fraction) (Fig. 3A). Although the net ^15^N allocation to proline underestimates the true net proline biosynthesis flux, it is still a reliable tool to compare net flux variations. We found that net ^15^N allocation to proline was significantly lowered from L15 to L11, L7, and L3, showing values of ~200 nmol ^15^N g^–1^ DW 4 h^–1^ in L15 to drop to 75 in L11 and then nearly 0 in L7/L3 (Fig. 3C). The results were consistent with a decrease of the net proline biosynthesis flux in source leaves. Such a decrease was strongly correlated with the decrease of *BnaP5CS1* transcript levels.

### Increase of *BnaA.ProDH2a* transcript levels in source leaves of *B. napus* does not correlate with variations of maximal proline degradation capacity

**Fig. 4.**
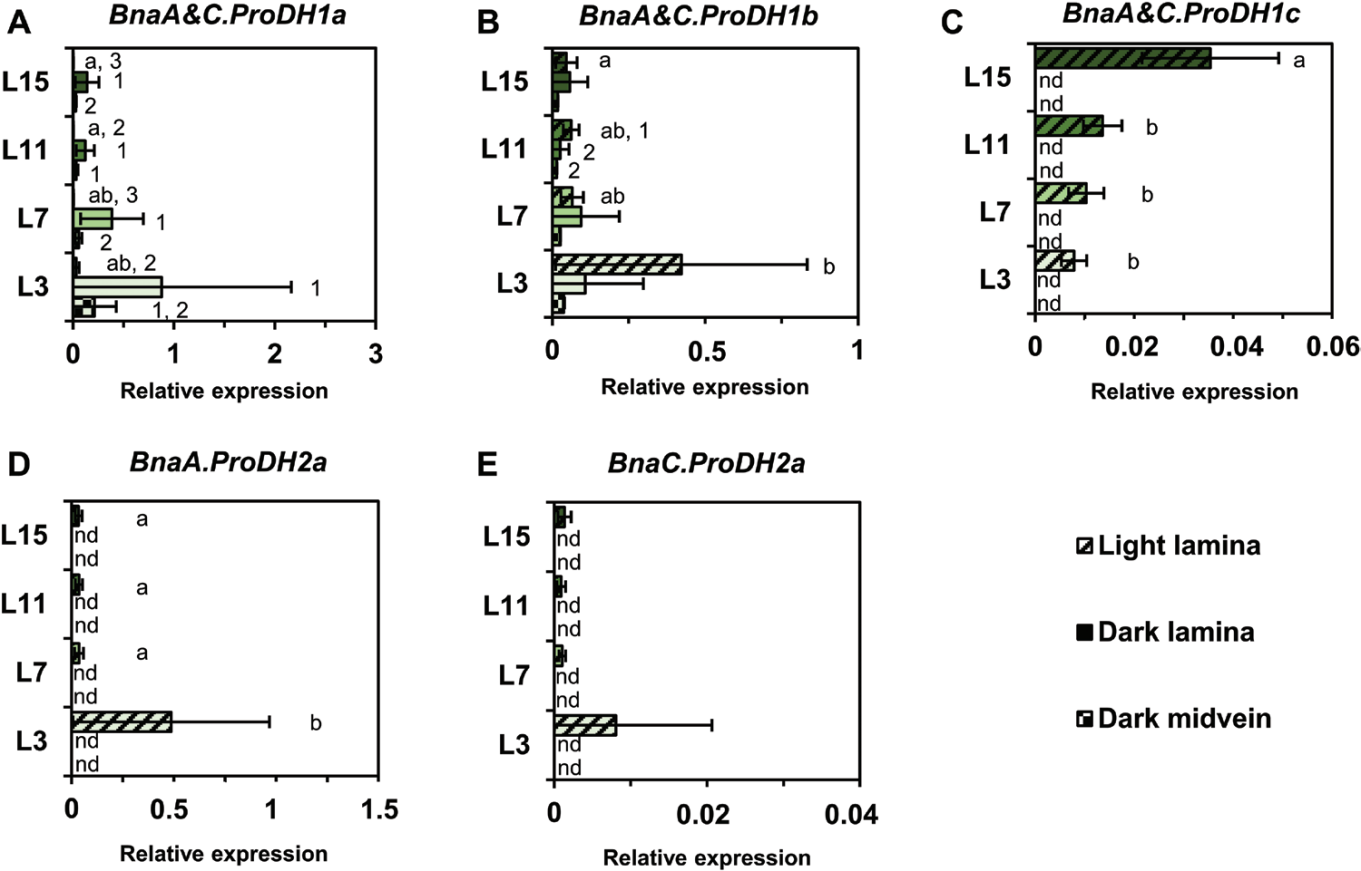
Two *ProDH* genes are overexpressed in source leaves of *B. napus*. Laminae from four leaf ranks having a different sink/source balance at the vegetative stage (60 DAS) were sampled either 3 h before (dark) or 3 h after (light) the beginning of the illumination period. In addition, the midveins sampled in dark conditions were also analysed. Expression of each gene copy was normalized relative to two reference genes *UBQ11* and *RibS3*. Values are the means ±SD of five independent biological replicates. Different letters indicate that mean values are significantly different between the different leaf ranks (*P*-value <0.05). Different numbers indicate that mean values are significantly different between the three conditions (light lamina, dark lamina, and dark midvein) for each leaf rank (*P*-value <0.05). (This figure is available in colour at *JXB* online.)

**Fig. 5.**
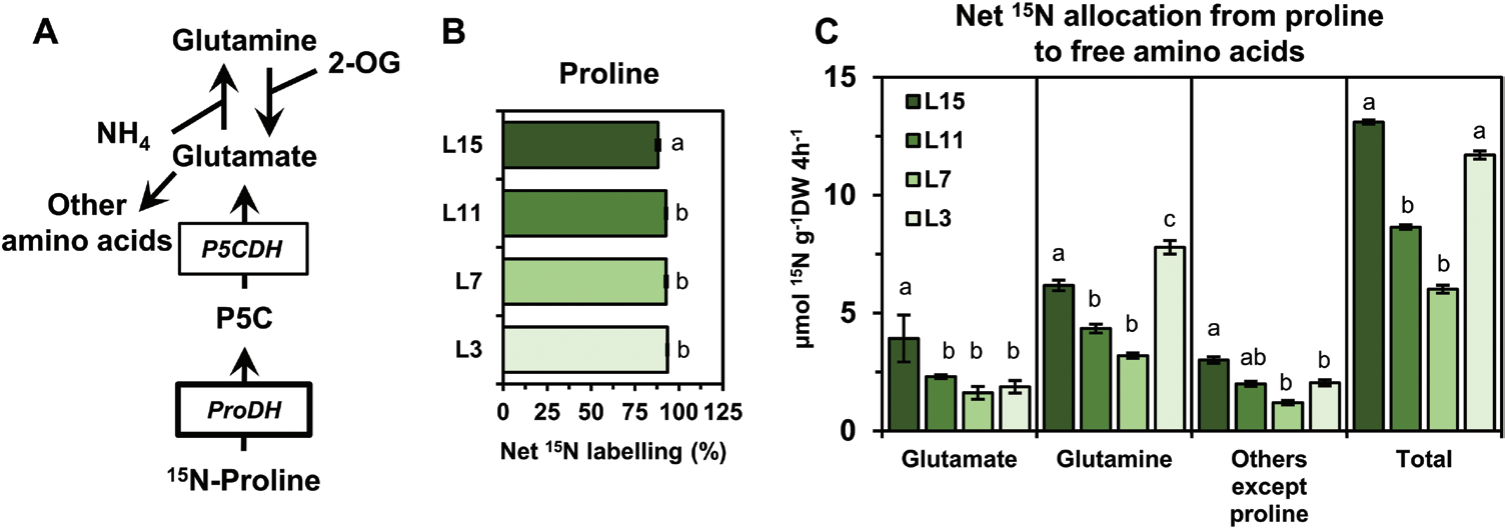
The variation of the maximal proline degradation capacity in leaves having a different sink/source balance poorly correlates with the variations of *BnaProDH* gene transcript levels. Leaf discs from four leaves having a different sink/source balance at the vegetative stage (60 DAS) were incubated for 4 h under continuous light with [^15^N]l-proline (98%). (A) Metabolic route for [^15^N]l-proline to amino acids. (B) Proline net ^15^N labelling. (C) Net ^15^N allocation from proline to free amino acids. ^15^N labelling detection and absolute quantification of proline were performed by MS and UV detection. Values are the means ±SD of three independent biological replicates. Different letters indicate that mean values are significantly different between the different leaf ranks (*P*-value <0.05). (This figure is available in colour at *JXB* online.)

After analysing the relationship between the net proline biosynthesis capacity and the transcriptional regulation of *P5CS* genes, we focused our work on the proline degradation pathway. Consequently, we quantified *ProDH* transcript levels with respect to dark–light transition and the sink/source balance of the leaves. Since the *BnaC.ProDH2a* gene has already been reported to be expressed in the midveins of senescent leaves (Faës *et al*., 2015) and as *ProDH* genes were overexpressed during dark periods in Arabidopsis (Hayashi *et al.*, 2000), we also analysed *BnaProDH* gene expression in the midveins of leaves sampled in the dark. Out of the six *BnaProDH1* genes, *BnaA&C.ProDH1a* and *BnaA&C.ProDH1b* genes were the most expressed and were significantly overexpressed in the lamina of leaves L7 and L3 compared with L15 (Fig. 4A–C). This overexpression was dependent on the light/dark condition (*BnaA&C.ProDH1a* genes for light, *BnaA&C.ProDH1b* genes for dark). *BnaA&C.ProDH1c* gene expression was significantly increased in leaves having a very low source status. However, those last genes were only detected in the lamina sampled in the light and were poorly expressed. Considering the two *BnaProDH2* genes, their expression levels were only detected in the lamina sampled in the light. The *BnaA.ProDH2a* gene was the most expressed and was significantly overexpressed in the lamina of the L3 senescing leaves (Fig. 4D, E). No *BnaProDH* gene was overexpressed in the midveins of leaves sampled in the dark compared with other conditions/tissues (Fig. 4). To summarize, *BnaA&C.ProDH1b* and *BnaA.ProDH2a* genes were overexpressed in leaves having a very strong source status, and showed a maximal expression value in the L3 senescent leaves. Since the net ^15^N allocation to proline was close to 0 in the L3 leaf rank (Fig. 3C), our results suggested that proline degradation could be activated in these tissues. Therefore, we also performed ^15^N labelling experiments on leaf discs isolated from the four leaf ranks using [^15^N]proline. This experiment aimed at comparing *in vivo* the maximal proline degradation capacity between the different leaf ranks, by measuring the incorporation of the ^15^N (resulting from proline) into other amino acids (Fig. 5A). After 4 h of [^15^N]proline labelling in isolated leaf discs, net ^15^N labelling in the proline molecule (M+1 isotopologue) was ~87–93% in the different leaf ranks (Fig. 5B). Although this enrichment was significantly lower in the L15 leaf rank compared with the other leaf ranks, the values were relatively similar. This suggested that [^15^N]proline might have been similarly incorporated in the leaf discs during the labelling experiment. From the net ^15^N labelling and quantification of amino acids, we calculated the net ^15^N allocation from proline to free amino acids (Fig. 5C). We found that net ^15^N allocation to glutamate and others amino acids (except proline) was significantly reduced from the leaf ranks L15 to L7. However, net ^15^N allocation to glutamine was significantly higher in L3 compared with other leaf ranks. Considering the total net ^15^N allocation from proline to free amino acids, our results showed that the maximal proline degradation capacity was decreased from L15 to L7, but was restored in L3 leaves (similarly to L15 leaves). Overall, our results suggest that proline catabolism is weakly activated in leaves having a very strong source status and may not be specifically controlled by variations of *BnaProDH* transcript levels.

### Some *BnaP5CS* and *BnaProDH* genes are specifically involved in development and stress response processes

We have shown that multiple copies of *P5CS* and *ProDH* genes were differentially expressed according to the sink/source status of the leaves in *B. napus*. Since the variation of *P5CS* and *ProDH* gene expression can control proline contents during some stress conditions, we decided to test whether specific *BnaP5CS* and *BnaProDH* genes were differentially expressed during multiple processes (sink/source status, dark to light transition, and stress response). Consequently, we analysed the gene-specific expression of *BnaP5CS* and *BnaProDH* genes during two stressing conditions known to regulate proline metabolism: an osmotic shock followed by a recovery period and a dark-induced senescence kinetic experiment (Trotel *et al.*, 1996; Dietrich *et al.*, 2011; Launay *et al.*, 2019). We decided to perform this experiment on the L7 leaf, since its source but not senescing leaf status allows a rapid entry into senescence during the dark-induced senescence experiment.

**Fig. 6.**
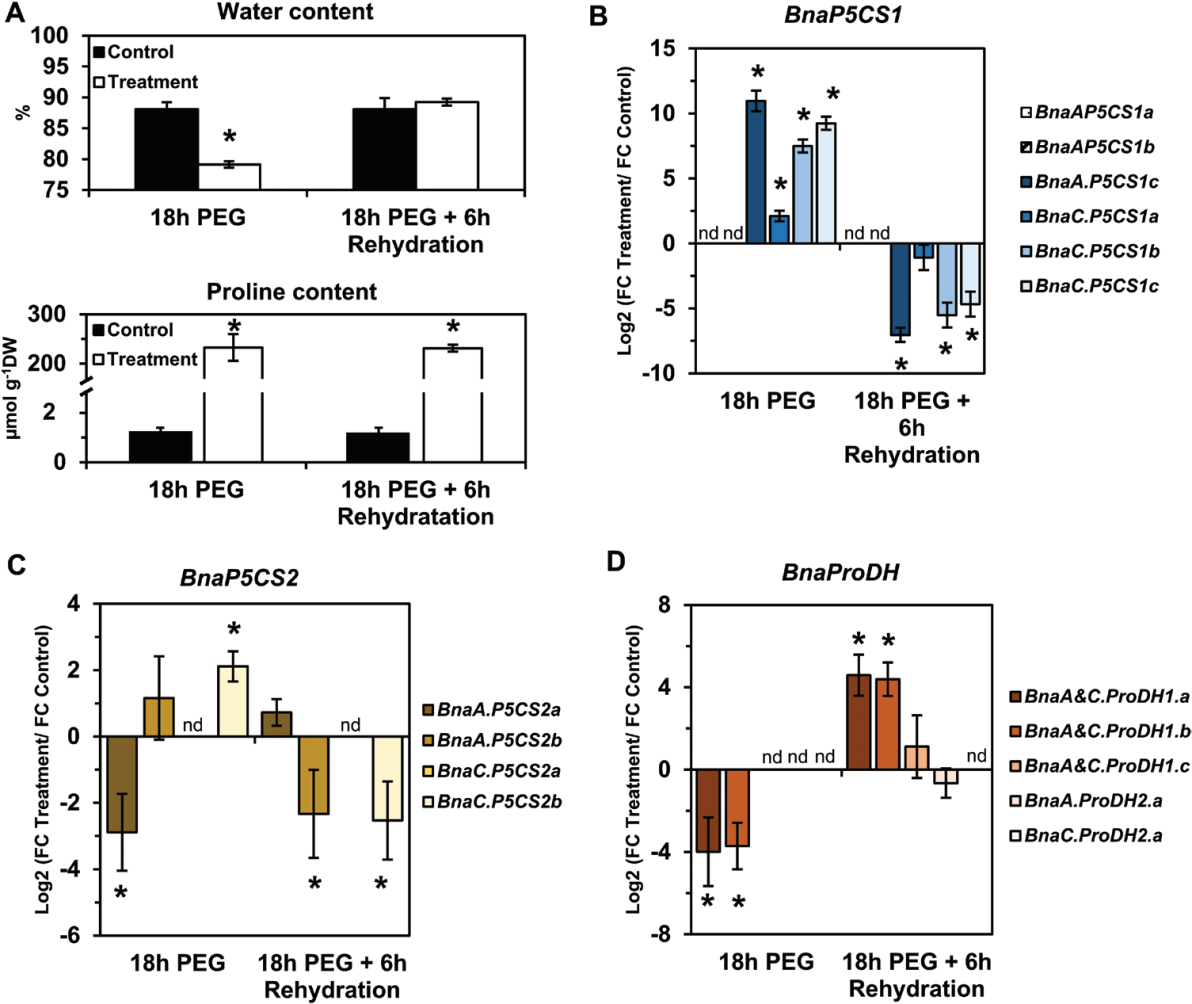
Transcript level variations of the *BnaP5CS* and *BnaProDH* genes during osmotic stress and rehydration in *B. napus*. For the ‘treatment’ condition, leaf discs from L7 leaves were subjected to a hyperosmotic stress (–1.88 MPa) for 18 h and then transferred to a hypo-osmotic medium for 6 h of rehydration under continuous light. For the ‘control’ condition, leaf discs from L7 leaves were incubated in the hypo-osmotic medium (same medium without PEG) for 18 h and then transferred to a new fresh medium for 6 h, under continuous light. (A) Water and proline content and log2 values for the ratio of fold change (FC) expression levels in the treated leaf discs over the control leaf discs for *BnaP5CS1* (B), *BnaP5CS2* (C), and *BnaProDH* genes (D). Values are the mean ±SD of three independent biological replicates. Asterisks indicate that mean values are significantly different between the two conditions (treatment versus control) (*P*-value <0.05). (This figure is available in colour at *JXB* online.)

**Fig. 7.**
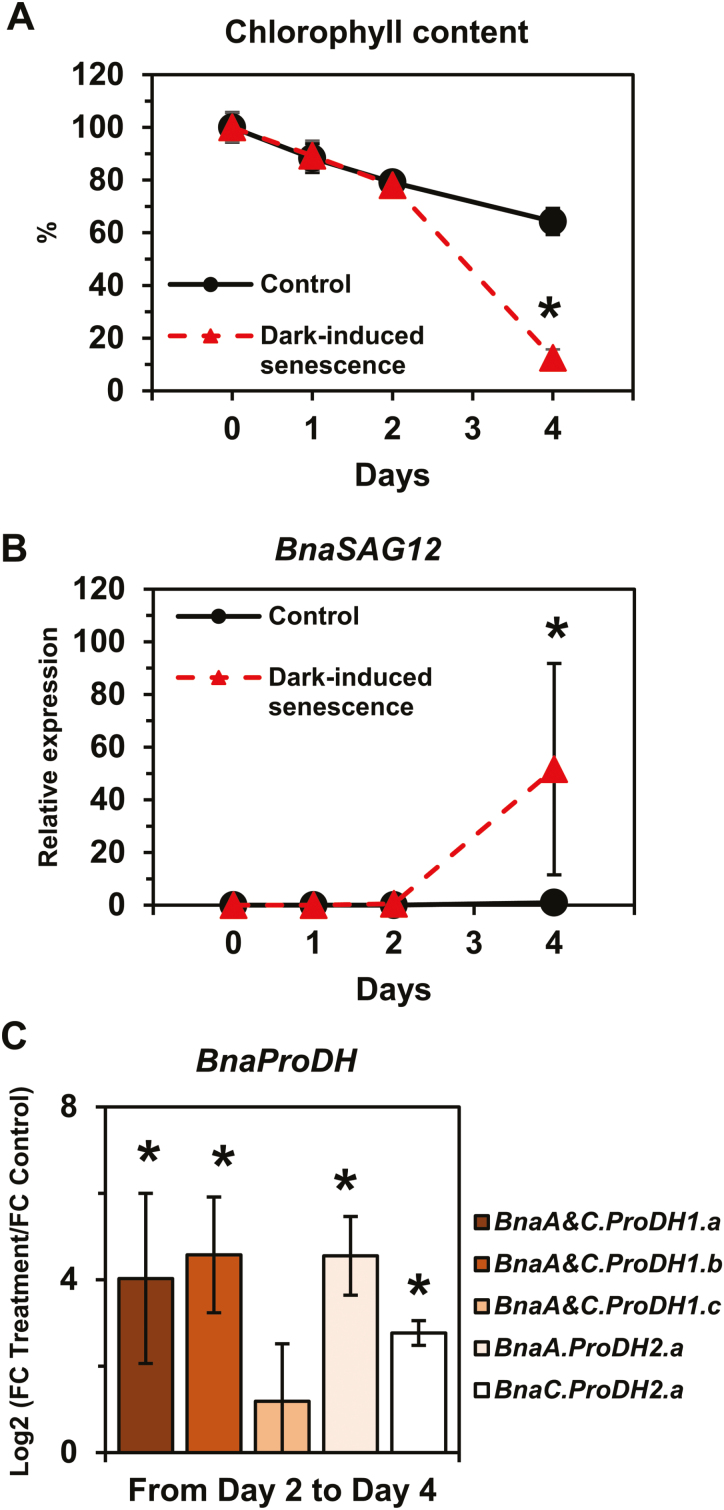
Dark-induced senescence triggers the expression of four *BnaProDH* genes in *B. napus*. Leaf discs from L7 were floated on water for 4 d and incubated under a light/dark cycle (14 h/10 h) (control) or under complete darkness (dark-induced senescence). (A) Chlorophyll content and (B) *SAG12* expression levels during the experiment. (C) Log2 values for the ratio of fold change (FC) expression levels in the treated leaf discs over the control leaf discs for *BnaProDH* genes from day 2 to day 4. Values are the mean ±SD of three independent biological replicates. Asterisks indicate that mean values are significantly different between the two conditions (treatment versus control) (*P*-value <0.05). (This figure is available in colour at *JXB* online.)

First, leaf discs from L7 were subjected to a PEG-induced hyperosmotic stress (–1.88 MPa) for 18 h under light conditions, followed by a 6 h recovery phase in a hypo-osmotic medium. The evolution of the leaf disc water content in the PEG treatment condition compared with the control condition confirmed that tissue dehydration occurred during hyperosmotic treatment whereas full tissue rehydration was restored after the recovery phase (Fig. 6A). A strong accumulation of proline was observed in the stressed tissues compared with the control after 18 h of PEG treatment. However, after 6 h of recovery, there was no significant decrease in proline content. Out of the four *BnaP5CS1* genes detected, the expression of the three genes *BnaA.P5CS1c*, *BnaC.P5CS1b*, and *BnaC.P5CS1c* was significantly highly induced (log2 FC variation >7) during the osmotic shock and was significantly repressed (log2 FC variation less than –4) during the post-stress recovery phase (Fig. 6B). *BnaC.P5CS1a* gene expression was moderately induced during the stress phase and repressed during the post-stress recovery phase. Surprisingly, we found that *BnaC.P5CS2b* gene expression was also moderately induced by the osmotic shock, whereas *BnaA.P5CS2a* gene expression was moderately repressed (Fig. 6C). During the recovery from hyperosmotic stress, *BnaA.P5CS2b* and *BnaC.P5CS2b* gene expression was significantly repressed. Regarding *BnaProDH* genes, only *BnaA&C.ProDH1a* and *BnaA&C.ProDH1b* gene expression was repressed during osmotic shock then induced during the recovery phase (Fig. 6D). Overall, our results demonstrated that three *BnaPCS1* genes were specifically involved in the control of proline content according to the sink/source balance of the leaves and during a dark–light transition and an osmotic stress and recovery. Next, we performed a time-course dark-induced senescence experiment on leaf discs from L7 to explore the differential expression of *BnaProDH* genes during stress-induced senescence processes. After 2 d of incubation, chlorophyll content was not significantly different in the control compared with the prolonged darkness condition (Fig. 7A). After 4 d, a significant decrease in chlorophyll content was observed in the prolonged darkness condition compared with the control. Concomitantly, transcription of the senescence marker genes *BnaA&C.SAG12-1&2* was strongly induced by prolonged darkness from day 2 to day 4 compared with the control (Fig. 7B). Those results confirmed that dark-induced senescence only operated from day 2 to day 4 in our experimental set-up. As a result, except for *BnaA&C.ProDH1c*, all *BnaProDH* genes were significantly induced (log2 FC variation >3) by dark-induced senescence compared with the control (Fig. 7C). Therefore, our results demonstrated that *BnaA&C.ProDH1a* gene expression was specifically controlled during a dark–light transition, an osmotic shock, recovery, and a dark-induced senescence, whereas *BnaA&C.ProDH1b* and *BnaA.ProDH2a* gene expression was specifically controlled by senescence (natural or dark induced).

## Discussion

Proline metabolism plays an essential role in plant adaptation to multiple environmental stress conditions, specifically when they are facing biotic and abiotic stresses (Szabados and Savouré, 2010). The tight control of proline content, generally achieved by regulating the *P5CS/ProDH* balance at the transcriptional level, allows the plants to tolerate the stress condition (Slama *et al.*, 2015). However, proline metabolism can also participate in plant developmental events, by contributing to proline accumulation and/or degradation during specific growth phases. In particular in reproductive organs and tissues with actively dividing cells, proline accumulation was shown to be stimulated (Mattioli *et al.*, 2009; Biancucci *et al.*, 2015). Conversely, at the vegetative stage, several piesces of evidence suggested that proline catabolism could be activated during leaf senescence and source leaf status acquisition in *B. napus* and could fuel mitochondrial respiration (Faës *et al*., 2015; Clément *et al*., 2018; Launay *et al.*, 2019). Such a phenomenon of metabolic reconversion of proline can be of functional interest for N recycling and nutrient remobilization processes through the phloem only if it is adequately fed with proline arising from neosynthesis or proteolysis.

In this study, we have shown that four *BnaP5CS1* genes were underexpressed in leaves having a very strong source status during the vegetative growth phase, whereas transcript levels of two *BnaProDH* genes were moderately overexpressed in the L3 senescent leaves (Figs 2B–E, 4B, D). These changes in transcript levels were strongly correlated with the depletion in proline content and with the reduction in commitment of *de novo* assimilated N towards proline biosynthesis, whereas maximal proline degradation capacity was not different between L15 (very low source status) and L3 (very strong source status) leaves (Figs 1H, 3C, 5C). We also found that transcription of three *BnaP5CS1* genes was strongly induced during an osmotic stress, and was repressed during the post-recovery phase (Fig. 6B). In particular, these changes were associated with strong variations of proline contents (Fig. 6A). In Arabidopsis, during an osmotic stress and the post-recovery phase, the modulation of proline content was associated with the overexpression of the *P5CS1* gene, whereas expression of the *P5CR* gene was not changed at all (Sharma and Verslues, 2010). Indeed, P5CR from Arabidopsis has a higher protein stability compared with P5CR from *Escherichia coli*, which may explain why this gene is less transcribed (Funck *et al.*, 2012). Therefore, our results suggest that the variations of *P5CS* transcript levels in *B. napus* may actively affect the biosynthesis of proline.

The specific control of proline content by the transcript levels of *BnaP5CS1* genes according to the sink/source status of the leaves may be explained by different physiological processes. First, growth and development of young leaves requires protein biosynthesis whereas old leaves having a very strong source status degrade their proteins and recycle their macromolecules. Increasing proline biosynthesis in young leaves compared with old source leaves could support the demand for proline to synthesize proteins in young leaves and to fuel high-energy-consuming cell division processes (Kavi Kishor and Sreenivasulu, 2014). Secondly, old leaves having a very strong source status are more hydrated than young leaves (Musse *et al.*, 2013; Sorin *et al.*, 2015). We have shown that the transcription of *BnaP5CS1* genes was decreased during tissue hydration after a hyperosmotic shock. Therefore, the decrease of net proline biosynthesis flux in source leaves may be a side effect of the tissue hydration. In addition to these factors, *BnaP5CS1* may also be down-regulated by other factors, including a crosstalk between senescence and the redox balance, regarding its up-regulation by light (our work; Hayashi *et al.*, 2000) and by the redox status (Aswani *et al.*, 2019).

We have identified that *BnaA&C*.*ProDH1a* genes were specifically overexpressed in dark conditions compared with light conditions (Fig. 4A), in good agreement with previous work performed in Arabidopsis (Hayashi *et al.*, 2000). *ProDH1* and *ProDH2* are located in mitochondria and can provide electrons to the mitochondrial electron transfer chain through proline degradation (Cabassa-Hourton *et al.*, 2016; Launay *et al.*, 2019). Since mitochondrial respiration is inhibited up to 95% by light in plants (Tcherkez *et al.*, 2005), we propose that *BnaA&C*.*ProDH1a* genes are involved in proline-dependent mitochondrial respiration. However, other *BnaProDH* genes are overexpressed in dark-induced senescence conditions and therefore could be involved in this proline-dependent respiration (Fig. 7C). Indeed, in Arabidopsis, some C starvation conditions {dark-induced senescence or [3-(3,4-dichlorophenyl)-1,1-dimethylurea] (DCMU)} can promote the expression of *ProDH1* (Dietrich *et al.*, 2011). Recently, the analysis of the double mutant *prodh1prodh2* in Arabidopsis confirmed that proline was effectively used as a substrate for mitochondrial respiration after 5 d of dark-induced senescence (Launay *et al.*, 2019). Our [^15^N]proline labelling experiments showed that the proline catabolism may be weakly activated between L7 and L3 leaves (i.e. between a source leaf and a source-to-senescent leaf) (Fig. 5C). In plants, many amino acids can be used as alternative respiratory substrates when C is missing (dark-induced senescence), such as leucine, isoleucine, valine, and tyrosine (Araujo *et al*., 2008, 2010, 2012, 2013; Chrobok *et al.*, 2016; Wang *et al.*, 2019). In our conditions, the overexpression of six *BnaProDH* genes during dark-induced senescence was observed in leaf discs containing very low chlorophyll contents (equivalent to 4 SPAD units) (Fig. 7C). Conversely, at an intermediary chlorophyll content in the L3 leaves (~20 SPAD units), only three *BnaProDH* genes were moderately overexpressed (Fig. 4A, B, D). Those observations suggest that the activation of proline catabolism in *B. napus* may be effective in leaves having a late stage of senescence, namely when the major part of the N content has been remobilized and other respiratory sources have already been depleted.

**Fig. 8.**
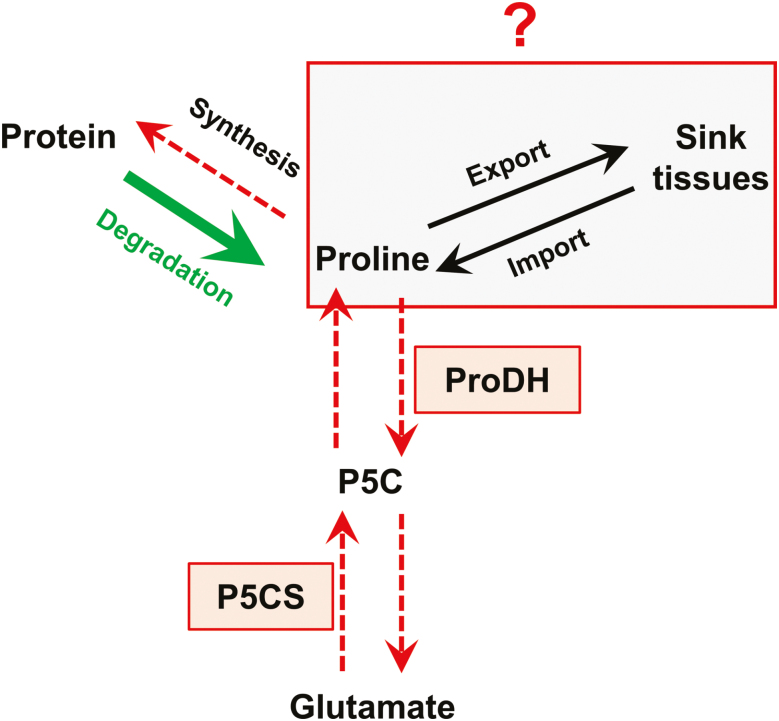
The regulation of proline content in source leaves of *B. napus* at the vegetative growth phase. Protein degradation in source leaves produces proline, which is not accumulated compared with leaves having a low sink/source balance. Our results showed that net proline biosynthesis flux was reduced in source leaves, whereas the maximal proline degradation capacity was weakly affected by the sink/source balance of the leaves. Consequently, proline arising from protein degradation could be exported from source leaves to organs having a low sink/source balance. Overall, our results suggest that proline catabolism plays a minor role in nitrogen remobilization processes in source leaves. (This figure is available in colour at *JXB* online.)

Our results showed that proline was not accumulated in source leaves and that proline catabolism was weakly activated according to the sink/source status of the leaves (Figs 1H, 5C). Consequently, proline arising from protein degradation in source leaves could be transported to young leaves and sink tissues through the phloem (Fig. 8). The source–sink apoplastic transport of amino acid through the phloem represents a key determinant of the N utilization efficiency for crops (Perchlik and Tegeder, 2017). In white clover, ^15^N tracing experiments showed that proline can be actively loaded to the phloem for transport between roots and shoots, suggesting that an equivalent transport between leaves having a different sink/source balance may also occur at the vegetative stage (Lee *et al.*, 2009). The differential proline enrichment found in phloem sap along the leaf ranks during drought stress also showed that an active source–sink proline transport can take place under stress conditions (Girousse *et al.*, 1996; Albert *et al.*, 2012). To date, no cell to cell proline transporter has been identified to play a role in proline remobilization at the vegetative stage in *Brassica* species. However, one proline-specific transporter (AtProT2) and one amino acid permease (AAP1) can both contribute to proline uptake under normal or stress conditions (Lehmann *et al.*, 2011; Wang *et al.*, 2017).

Concerning the sub-/neofunctionnalization of proline metabolism-associated gene copies, we have shown that some *BnaP5CS1* and *BnaProDH* genes shared conserved multiple functions in *B. napus*. The expression of three out of six *BnaP5CS1* genes was controlled by both the sink/source status of the leaf and osmotic stress (Figs 2B, D, E, 6B), and the expression of four out of six *ProDH1* genes was controlled by both osmotic stress and dark-induced senescence (Figs 6D, 7C). Since parental ancestors of oilseed rape have undergone a whole-genome triplication event, the presence of multiple copies was expected to relax natural and artificial selective pressures on any individual gene copy, thereby facilitating the emergence of novel gene function (Conant and Wolfe, 2008). In the oilseed rape genome, duplicated flowering time genes were shown to be preferentially retained compared with other genes, and ~70% of them had a divergent expression patterns relative to each other across development, suggesting neo- or subfunctionalization (Jones *et al.*, 2018). Such conserved multifunctionalization of *P5CS* and *ProDH* genes in *B. napus* clearly reflects the importance of finely controlling both phases of proline metabolism (biosynthesis and degradation) to face issues related to both development and stress conditions.

In conclusion, we have shown that the depletion of proline content in source leaves in *B. napus* was associated with a decrease in the net proline biosynthesis flux rather than an activation of proline catabolism. Such flux variations were correlated with the strong underexpression of four *BnaP5CS1* genes, whereas the expression of three *BnaDH* genes was moderately increased in source leaves to senescent leaves. Since *BnaProDH* genes were strongly overexpressed during dark-induced senescence (C starvation condition), our results suggested that proline catabolism may played a role in *B. napus* leaves but during late stages of senescence, perhaps to contribute to proline-dependent mitochondrial respiration. Consequently, the proline arising from protein degradation in source leaves might be remobilized toward young leaves and sink tissues through the phloem. Recent works showed that N use efficiency and seed yields could be dramatically increased in crops by enhancing the expression of the amino acid transporter AAP1, which can contribute to proline uptake (Perchlik and Tegeder, 2017; Wang *et al.*, 2017). Future research should focus on the possible source–sink proline transport at the vegetative stage to identify potential new targets for improvement of N use efficiency.

## Supplementary data

Supplementary data are available at *JXB* online.

**Fig. S1.** FW and DW per leaf area and FW/DW ratio of the four leaf ranks having a different sink/source balance (L15, L11, L7, L3).

**Fig. S2.** Molecular phylogenetic analysis of the *Brassica napus P5CS* coding sequences.

**Fig. S3.** Relative expression for each *BnaP5CS2* gene in four leaf ranks having a different sink/source balance (L15, L11, L7, L3) harvested under either dark or light conditions.

**Table S1.** Amino acid contents in the laminae of four leaf ranks having a different sink/source balance (L15, L11, L7, L3) sampled 3 h after the beginning of the illumination period.

**Table S2.** List of primers used in this study for qPCR analysis and primer efficiency.

erz538_suppl_supplementary_Materials
