# Supplementary material for: Leaf status and environmental signals jointly regulate proline metabolism in winter oilseed rape
Source: J Exp Bot. 2019 Dec 6;71(6):2098–111. doi: 10.1093/jxb/erz538 (PMC7242077; doi:10.1093/jxb/erz538)
Supplement: erz538_suppl_supplementary_Materials [file erz538_suppl_supplementary_materials.pdf]

Supplementary Material

Supplementary fig. S1. FW and DW per leaf area and FW/DW ratio of four leaf ranks having a different sink/source balance (L15, L11, L7, L3). Values are means ± SD (n = 5 independent biological replicates). Different letters indicate that mean values are significantly different (p-value < 0.05) between the different leaf ranks.

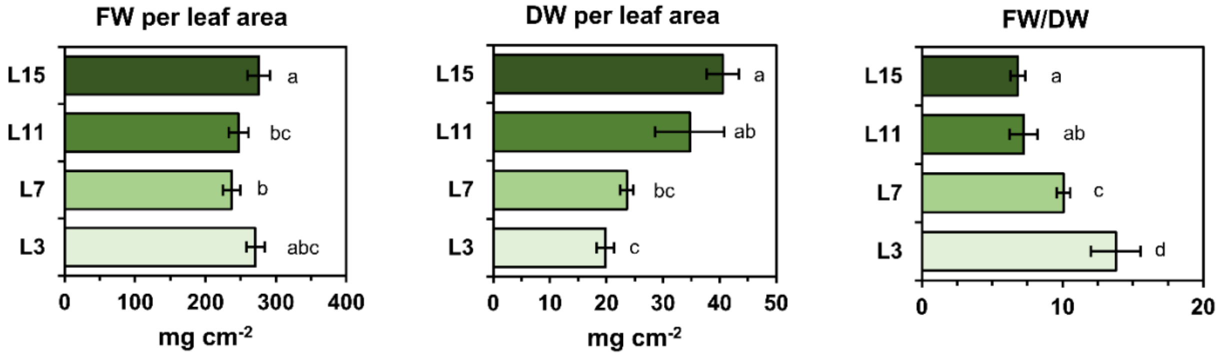

Supplementary fig. S2. Molecular phylogenetic analysis of the *Brassica napus* P5CS coding sequences. Sequence alignments were performed with MUSCLE, phylogenetic analysis with the maximum likelihood method and the nucleotide substitution model of Tamura-Nei, assuming uniform rates among sites (MEGAX software). The consensus tree from 1000 bootstrap replicates is shown with bootstrap values expressed between 0 and 100.

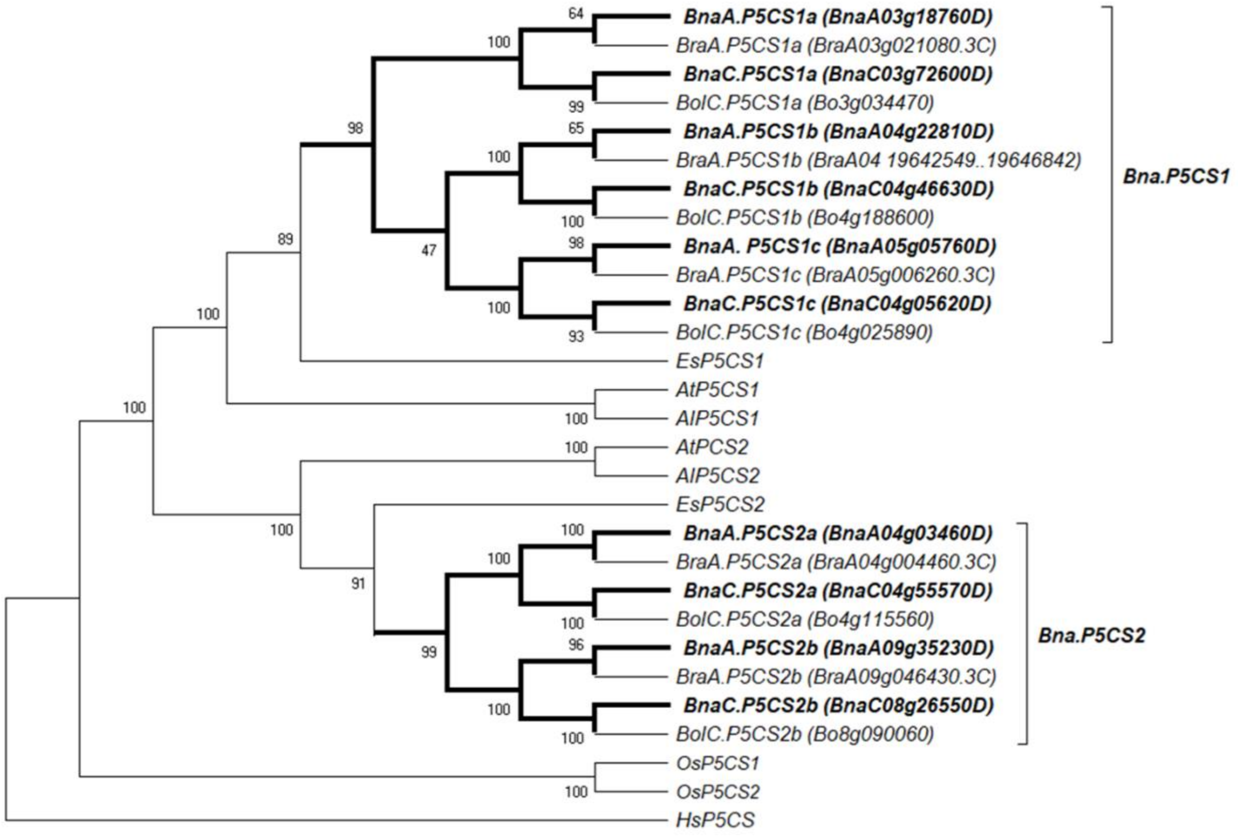

Supplementary fig. S3. Relative expression for each *BnaP5CS2* gene in four leaf ranks having a different sink/source balance (L15, L11, L7, L3) harvested either under dark or light conditions. Expression of each gene copy was normalized relatively to two reference genes *UBQ11* and *RibS3*. Values are the mean ± SD of 5 independent biological replicates (nd = not detected). Different letters indicate that mean values are significantly different (p-value < 0.05) between the different leaf ranks. Different numbers indicate that mean values are significantly different (p-value < 0.05) between the dark and light conditions for each leaf rank.

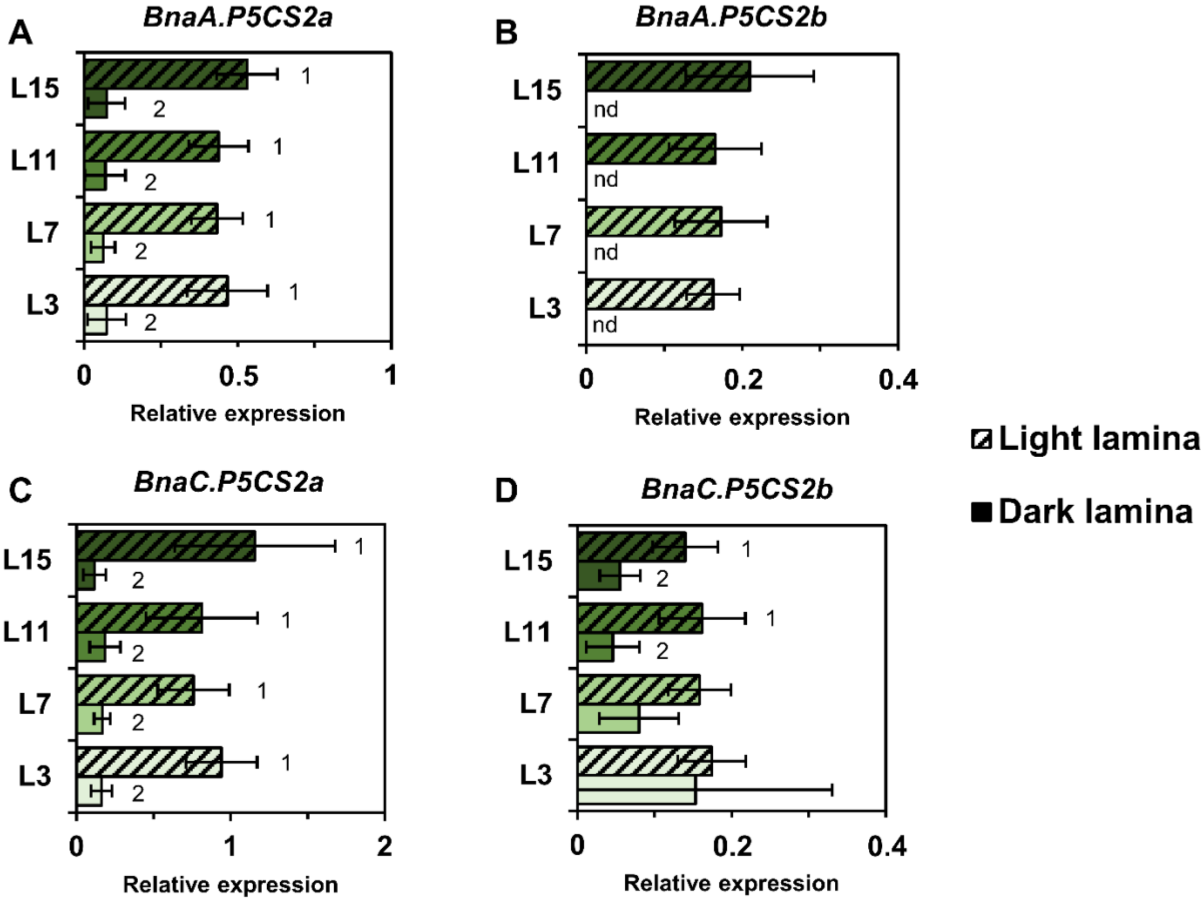

Table S1. Content of amino acids detected in the laminae of four leaf ranks having a different sink/source balance (L15, L11, L7, L3) sampled 3 hours after the beginning of the illumination period. Values are means ± SD (n = 5 independent biological replicates), expressed in µmol g^{-1}DW (nd = not detected). Different letters indicate that mean values are significantly different (p-value < 0.05) between the different leaf ranks. *SMC = S-methylcysteine*; *SMCSO = S-methylcysteine sulfoxide.*

**Leaf ranks**	**L15**		**L11**		**L7**		**L3**	
	**Mean**	**SD**	**Mean**	**SD**	**Mean**	**SD**	**Mean**	**SD**
**Alpha-Alanine**	1,46 a	0,24	1,06 ab	0,34	0,96 b	0,23	0,74 b	0,16
**Arginine**	0,18 a	0,02	0,21 a	0,11	0,19 a	0,04	0,05 b	0,07
**Asparagine**	0,91 a	0,32	0,44 b	0,29	0,53 ab	0,23	0,14 b	0,15
**Aspartate**	7,77 a	1,24	3,80 bc	0,52	4,99 b	1,41	2,95 c	0,83
**Beta-Alanine**	0,07 a	0,01	0,03 b	0,04	nd		nd	
**Glutamate**	14,52 a	1,43	10,98 ac	2,45	15,43 ab	3,15	10,18 c	1,12
**Glutamine**	14,81 a	5,65	4,47 b	3,02	4,65 b	2,02	2,20 b	0,67
**Glycine**	0,23 a	0,03	0,38 ab	0,16	0,49 b	0,11	0,45 b	0,12
**Histidine**	0,00	0,00	0,11	0,08	nd		nd	
**Isoleucine**	0,38 a	0,06	0,27 ab	0,09	0,26 b	0,03	0,23 b	0,07
**Leucine**	0,10 a	0,02	0,25 b	0,10	0,28 b	0,05	0,29 b	0,10
**Lysine**	0,14 a	0,01	0,22 ab	0,08	0,26 b	0,03	0,26 b	0,07
**Methionine**	0,12 a	0,02	0,06 b	0,03	0,03 b	0,04	nd	
**SMC**	0,11	0,04	0,13	0,05	0,06	0,08	nd	
**Phenylalanine**	0,19	0,03	0,23	0,04	0,24	0,03	0,28	0,12
**Proline**	1,57 a	0,36	0,78 bc	0,12	0,91 b	0,18	0,52 c	0,06
**Serine**	6,29 a	1,59	5,41 ab	2,51	7,05 a	3,04	2,14 b	1,56
**SMCSO**	12,14 a	2,57	7,74 b	0,92	10,75 ab	1,71	8,56 b	1,30
**Threonine**	1,71 ac	0,40	1,68 ac	0,80	2,25 ab	0,67	1,15 bc	0,22
**Tryptophan**	0,00	0,00	0,10	0,03	0,04	0,04	0,06	0,09
**Tyrosine**	0,06 a	0,04	0,15 b	0,05	0,18 b	0,04	0,15 b	0,05
**Valine**	0,53	0,10	0,63	0,28	0,75	0,11	0,50	0,11

Table S2. List of primers used in this study for qPCR analysis and primers efficiency.

Gene name	forward	reverse	Primers efficiency
BnaA.P5CS1a (BnaA03g18760)	TCAACTTCTGGTGAATGATAGCAGT	TAAGGAGCTCTTCTGGTGCTGA	1,81
BnaA.P5CS1b (BnaA04g22810)	CGTTGAAGTTGTAGATGATGTG	TTGTACTTGCGTTGTGGAAT	1,93
BnaA.P5CS1c (BnaA05g05760)	GTGGGGATAAGCACAGGG	CCATCCTCTCCTAGTCTCC	2,14
BnaA.P5CS2a (BnaA04g03460)	ATAAGCACAAGCAGAATACAC	GAATCCGTTCTCCACTGCC	2,02
BnaA.P5CS2b (BnaA09g35230)	GATGCACATTTGTGGGCTAGA	GCTTCTAGAGCGTTGGCTATATCT	2,07
BnaC.P5CS1a (BnaC03g72600)	GAACAATGGAGTCACTTTGTAC	AATGTGATCAATAGCAGCATAG	2,14
BnaC.P5CS1b (BnaC04g46630)	GATTTAGAGCAGAACGCTGA	AGTACTCATGCTTGAACGAAG	2,01
BnaC.P5CS1c (BnaC04g05620)	CAGTGGGAGTGGAGGGAC	CCACAAAGAGACTTCACACAG	2,07
BnaC.P5CS2a (BnaC04g55570)	TCACCAAGAACGTTAAGCGCA	CTCAAATCCATCCGAGTTCAGT	1,99
BnaC.P5CS2b (BnaC08g26550)	TGCACATCTGTGGGCTCG	CTCATTTGCTTCAAGGGCTG	2,11
BnaA&CProDH1.a	ACGCACGATTGCTACAATA	AAACTCTATCTTCCCGTTCTC	2,06
BnaA&CProDH1.b	AGCAGCAAAATCCTTACCT	CTTCCACGAGAGTTTGAAAT	2,06
BnaA&C.ProDH1.c	ATTGAAGCTGCCAAGTCC	GTTCCCAACGAAGCAAGT	2,07
BnaA.ProDH2.a	GTGAGGAGCGTGTATGAGTC	ACAAATGGCTGTGATCTTAAC	1,94
BnaC.ProDH2.a	CGTGAGGAGTGTGTATGAGTT	GACAAATCGCTGTGATCTTAAT	1,97
BnaA&C.SAG12-1&2	GTTTTGTTTAGCCAAAGTCAAACA	CGGCGGAAGATTGGCT	2,01
BnaCab	GGCAGCCCATGGTACGGATC	CCTCCTCGCTGAAGATCTGT	2,11
BnaC.RibS3 (BnaC04g13040D)	CCTGTCGCCCAGAAGAAG	AGAGCCGCTGATAAGGTTTG	2,15
BnaC.UBQ11 (BnaC04g09150D)	GTTGATCTTCGCTGGAAAAC	CCATTAAAGACGGCTCGATG	2,10
